# Child Posture and Belt Fit in a Range of Booster Configurations

**DOI:** 10.3390/ijerph17030810

**Published:** 2020-01-28

**Authors:** Monica L.H. Jones, Sheila Ebert, Miriam A. Manary, Matthew P. Reed, Kathleen D. Klinich

**Affiliations:** University of Michigan Transportation Research Institute, University of Michigan, Ann Arbor, MI 48109, USA; ebertshe@umich.edu (S.E.); mmanary@umich.edu (M.A.M.); mreed@umich.edu (M.P.R.); kklinich@umich.edu (K.D.K.)

**Keywords:** children, belt fit, booster, belt positioning

## Abstract

Belt positioning boosters reduce injury risk for child occupants compared with seat belts alone. While boosters shorten the effective seat length (and thus reduce slouching), “boosting” the child relative to the vehicle interior components also achieves additional safety benefits. First, the increase of the lap belt angle usually improves belt fit across the pelvis and reduces the risk of the occupant slipping (“submarining”) under the belt. Second, the torso belt is re-centered over the bony landmarks of the shoulder for more effective/secure restraint. Third, the child’s head is relocated in a range better protected by side airbags. The objective of this research was to quantify differences in posture and belt fit across a range of booster designs that provide different levels of boosting. Posture and belt fit were measured in 25 child volunteers aged four to 12. Children were measured in three laboratory seating conditions selected to provide a range of cushion lengths and belt geometries. Six different boosters, as well as a no-booster condition, were evaluated. The low height boosters produced postures that were more slouched, with the hips further forward than in other more typical boosters. Lap belt fit in the low height boosters was not meaningfully different from the other boosters. Shoulder belt fit produced by the lowest height booster was similar to the no-booster condition. Belt positioning boosters that boost the child less than 70 mm produced postures similar to the no-booster condition. While lap belt guides on these products can produce a similar static lap belt fit, they may not provide adequate dynamic performance and do not achieve the other benefits that come with raising the child to a more advantageous location relative to interior components and belts.

## 1. Introduction

Belt positioning boosters are recommended for use by children who have outgrown a forward-facing harnessed child restraint system (CRS), but are too small to achieve good belt fit using a vehicle lap-shoulder belt. The latest national estimates of booster use in the United States indicate that 45% of children aged four to seven use boosters [1]. A prior study on belt fit showed that most children under age 11 would benefit from using a booster to achieve a good belt fit [2]. Field data indicated that children aged 4–10 using boosters were 30–45% less likely to sustain injury in a crash compared to children using seat belts alone [3,4,5]. 

Several studies of child posture and belt fit have identified several effectual ways boosters reduce injury risk [6,7,8,9,10,11,12,13,14,15]. First, since standard vehicle safety features (including the seat belt system, airbags, and interior padding) are nominally designed for adults, most booster-age children cannot reap the benefits offered by the safety features. By repositioning the child’s body upward, the booster allows a child occupant to achieve a skeletal position similar to that of an adult. Historically, products have achieved this by boosting the child’s seated position upward by at least 75–100 mm. The vertical shift re-positions the child so that the lap belt sits at the top of the thigh close to the pelvic bone. Meanwhile, the shoulder belt crosses the clavicle, the center of the sternum, and the opposite hip near the greater trochanter of the femur. The resulting seat belt fit creates an advantageous side view lap belt angle that prevents the lap belt from shifting upward during a crash. Second, boosters may have lap and shoulder belt guides to help establish and maintain good belt fit for the entire duration of travel. Third, a booster that raises the child effectively shortens the cushion length, allowing them to sit more upright and to maintain a comfortable bent leg posture throughout a trip. This modified posture also contributes to better belt fit.

Some new boosters have reduced thickness compared with conventional boosters and consequently raise the child’s body upward to a lesser amount. Boosters that provide a minimum level of “boost” to anthropometric testing devices (ATD) have not been considered in previous studies. This boost is measured by the difference in the height of the ATD H-point (hip joint center) with and without the booster. In this context, the purpose of this research project was to measure the posture and belt fit of children and ATD in a range of boosters that included these new designs.

## 2. Materials and Methods

### 2.1. Booster Selection

Booster evaluation commenced with review of the 2017 list of boosters that was compiled on the American Academy of Pediatrics website [16]. The range of styles listed included backless (cushions), highback-to-backless, highback only, 3-in-1, and combination. The booster ratings issued by the Insurance Institute for Highway Safety (IIHS) were also reviewed [17]. We chose to consider products that did not receive this rating; specifically, we selected one product rated as “not recommended”, two products rated as “good bet”, and one product rated as “check fit”. Since one of the objectives of this study was to examine the posture of children in boosters that did not “boost” as much as traditional booster products, we also reviewed product photos and evaluated three lower height products. One was rated as “best bet” by the IIHS and two were not rated by IIHS. The remaining four booster products (all rated as “best bets”) were selected to fill in the distribution of booster styles and heights while trying to vary manufacturers and belt routing features.

For this study, we chose to characterize each booster by quantifying the level of “boost” and belt fit scores. Due to the widely varying shapes and contours of boosters, geometric measurement was not a consistent way to define the amount by which a booster might elevate a child. Instead, boost was quantified by the difference in the height of the Hybrid III 6-year-old (YO) ATD H-point seated on the Federal Motor Vehicle Safety Standards (FMVSS) No. 213 bench with and without a booster [18]. This measurement takes into account the compression of both the booster and the test bench. The effects of the booster geometry on the seated posture and position of the ATD were also accounted for. Subsequently, this measure was chosen as the best estimate of the typical effect the booster might have in a vehicle on the height of the child’s pelvis. 

Belt fit measurements were also performed using the Hybrid III 6YO ATD. For each booster configuration, lap belt and shoulder belt fit were quantified in a laboratory rear seat assembly used in a previous University of Michigan Transportation Research Institute (UMTRI) study of adult passenger posture and belt fit [19]. This mockup has a cushion with minimal bolstering and a length of 465 mm. Adjustable lower belt anchorage locations produced 30 and 75 degree lap belt angles according to the FMVSS 210 definition (19.49 CFR § 571.210) [20]. Belt fit was measured with a total of four belt geometries by combining the two lap belt angles with two shoulder-belt upper anchorage locations.

Using previously published methods [12], lap belt fit was quantified relative to the projection of the anterior superior iliac spine (ASIS) of the ATD pelvis bone onto the surface of the ATD skin. The lap belt score (LBS) was computed as the distance below or forward of the ASIS at the upper or rearward edge of the belt [12]. LBS score is defined along the side-view profile of the pelvis and thighs at the lateral position of the ASIS. Right and left side measures were averaged. The shoulder belt score (SBS) was quantified as the lateral position relative to the body centerline at the height of the bottom of the neck [12].

**Figure 1:**
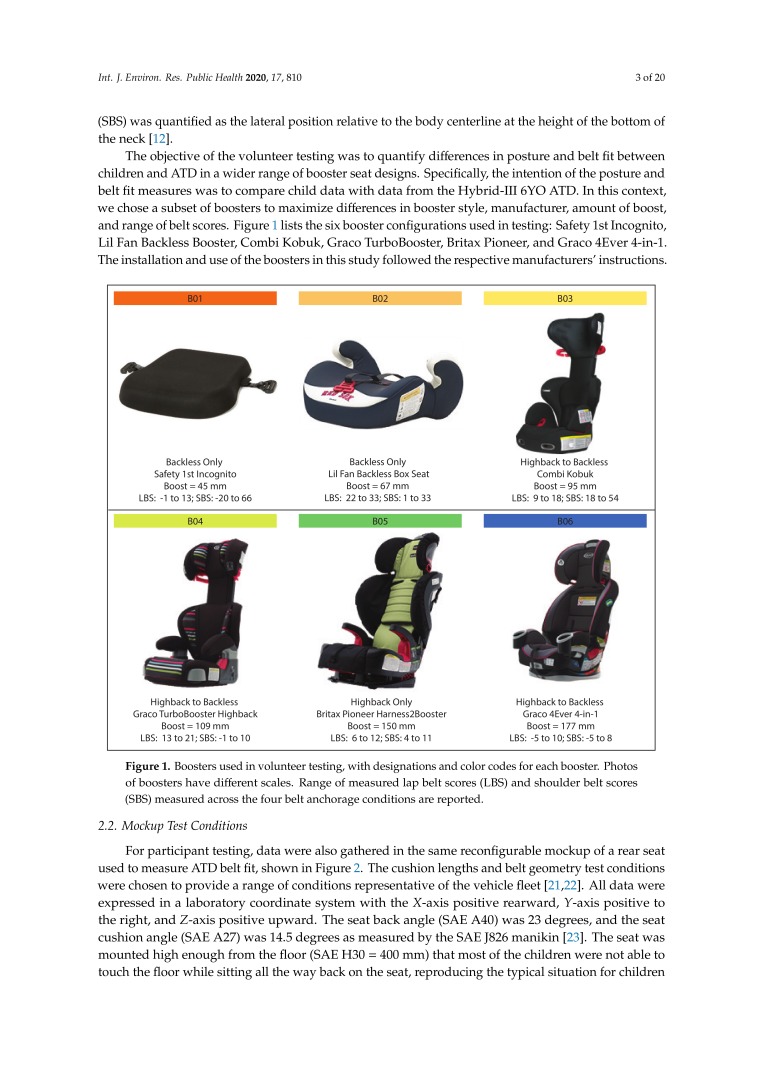
Boosters used in volunteer testing, with designations and color codes for each booster. Photos of boosters have different scales. Range of measured lap belt scores (LBS) and shoulder belt scores (SBS) measured across the four belt anchorage conditions are reported.

The objective of the volunteer testing was to quantify differences in posture and belt fit between children and ATD in a wider range of booster seat designs. Specifically, the intention of the posture and belt fit measures was to compare child data with data from the Hybrid-III 6YO ATD. In this context, we chose a subset of boosters to maximize differences in booster style, manufacturer, amount of boost, and range of belt scores. Figure 1 lists the six booster configurations used in testing: Safety 1st Incognito, Lil Fan Backless Booster, Combi Kobuk, Graco TurboBooster, Britax Pioneer, and Graco 4Ever 4-in-1. The installation and use of the boosters in this study followed the respective manufacturers’ instructions. 

### 2.2. Mockup Test Conditions

**Figure 2:**
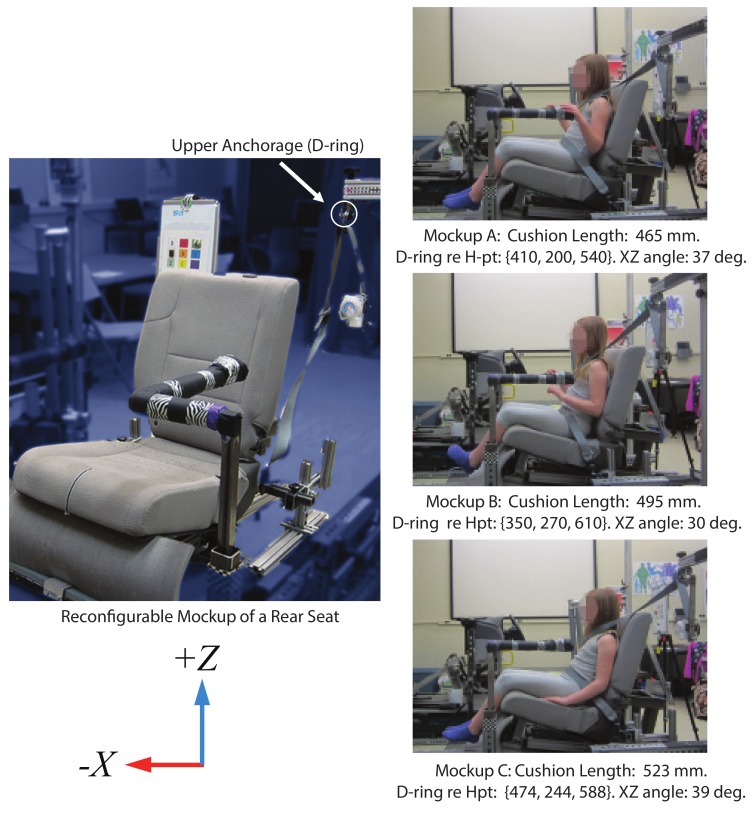
Reconfigurable mockup of a rear seat. The upper anchorage (D-ring) location can be adjusted on all three axes, and the lower anchorage can be adjusted fore-aft. Cushion length test conditions on the mockup are illustrated.

For participant testing, data were also gathered in the same reconfigurable mockup of a rear seat used to measure ATD belt fit, shown in Figure 2. The cushion lengths and belt geometry test conditions were chosen to provide a range of conditions representative of the vehicle fleet [21,22]. All data were expressed in a laboratory coordinate system with the *X*-axis positive rearward, *Y*-axis positive to the right, and *Z*-axis positive upward. The seat back angle (SAE A40) was 23 degrees, and the seat cushion angle (SAE A27) was 14.5 degrees as measured by the SAE J826 manikin [23]. The seat was mounted high enough from the floor (SAE H30 = 400 mm) that most of the children were not able to touch the floor while sitting all the way back on the seat, reproducing the typical situation for children in rear vehicle seats. Three mockup test conditions were evaluated, distinguished by seat cushion lengths of 465, 495, and 523 mm. Seat cushion length was measured using a tool that was developed in Huang and Reed [21] to approximate the SAE corresponding J2732 dimension. The cushion length test conditions were selected to represent the median, mean, and maximum from the Huang and Reed study [21].

Seat belt anchorage locations documented in the rear seat [22], as well as the belt anchorage conditions used by IIHS in assessing belt fit (IIHS 2018) were reviewed. Table 1 summarizes the belt anchorage locations and angles selected for use with the reconfigurable mockup relative to the fleet measurements reported by Reed and Ebert [22]. Upper anchorage (shoulder belt D-ring) location was quantified using side-view (*XZ*) and front-view (*YZ*) angles of the vector from the seat H-point on the centerline to the D-ring bolt [15]. Mockup Condition C had a belt geometry that matched the geometry of a modified FMVSS No. 213 seat assembly [24]. Geometries for Mockup Conditions A and B were selected to produce *XZ* and *YZ* angles that spanned the range most commonly found in the U.S. passenger vehicle fleet [22]. 

### 2.3. Participants

**Figure 3:**
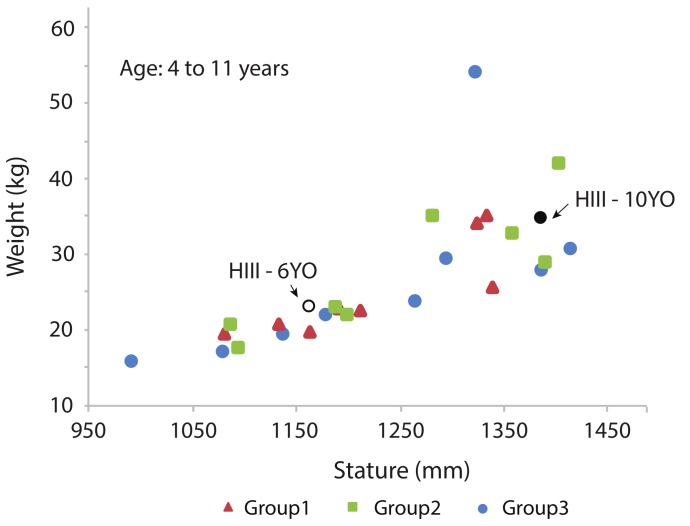
Stature and body weight participant pool (12 girls and 13 boys) along with reference stature and weight for the Hybrid-III 6YO and 10YO anthropometric testing devices (ATD) [25].

Twenty-five children (12 girls and 13 boys) between the ages of four and 12 years were recruited to participate in the study. Volunteers were recruited by word of mouth, online advertising, and the University of Michigan Human Research Recruiting Registry website (http://www.UMHealthResearch.org). The goal was to recruit children who spanned the range of potential users of belt positioning boosters with respect to stature and weight. This span included the range of the 6YO and 10YO Hybrid-III crash dummies. Participants were then assigned to three groups. The objective was for each group to have similar distributions in terms of sex, stature, and weight distribution. Each participant was tested in each mockup test condition: one in the no-booster condition and in two different boosters assigned to their group (a total of three conditions per participant). The weight-by-stature distribution of child participants for each group is shown in Figure 3 and Table 2. All test procedures were approved by the Institutional Review Board at the University of Michigan (HUM00123548). Written informed consent was obtained from the parent or guardian of each participant; each child assented orally.

### 2.4. Participant Body Dimensions

**Figure 4:**
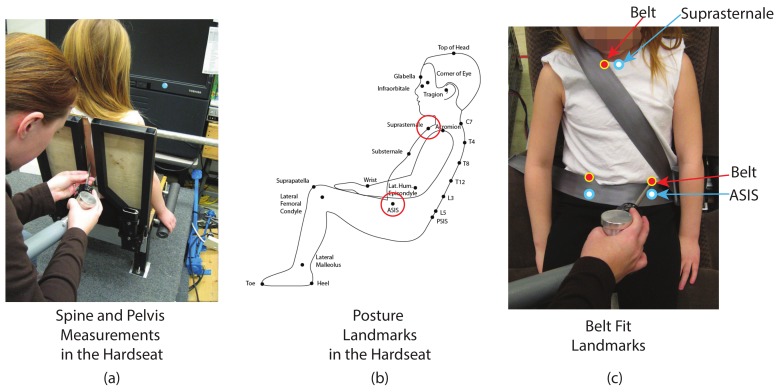
(**a**) Laboratory hardseat used to obtain posterior and anterior body landmarks in the same posture. (**b**) Schematic depiction of body landmarks that were digitized in the hardseat. (**c**) Digitizing lap and shoulder belt fit landmarks. ASIS, anterior superior iliac spine.

Each participant changed into test garments (a t-shirt and form-fitting leggings) provided by the investigators. The t-shirt was open in the back to facilitate access to posterior landmarks. Standard anthropometric dimensions (including stature, body weight, and linear breadths and depths) were gathered from each participant to characterize the overall body size and shape. Key landmarks were marked on each participant using washable markers. Body landmark locations were recorded (“digitized”) using a FARO Arm coordinate measurement machine (FARO Technologies, Lake Mary, FL). Each participant sat in a specially constructed laboratory hardseat that provided access to posterior landmarks on the spine and pelvis (Figure 4). The investigator, using the FARO Arm, digitized the landmarks while the child held a relaxed, sagittally symmetric posture. Figure 4 shows the landmarks schematically. The landmarks were digitized in several overlapping sets. If the child moved appreciably during a set, the set was replaced. The data from each set were aligned to the first set using the repeated points. These digitized surface landmarks were then used to compute the three-dimensional position and orientation of the pelvis. 

### 2.5. Test Protocol

Data collection was conducted using the posture and belt fit measurement protocols that have been used in several previous UMTRI studies of both child and adult posture and belt fit [12,26]. Child posture and belt fit were measured as each child sat in the selected boosters and assigned mockup test conditions. Child participants chose their own posture and donned the belt themselves. Once the participant sat approximately motionless, the investigator palpated the accessible body landmarks on the head, chest, pelvis, and extremities and recorded the landmark locations using the FARO Arm. The child was seated in each condition less than five minutes prior to measurement.

The FARO Arm was also used to collect landmarks on the belt and streams of data to define the belt path. A close-up of the points used to document lap belt position relative to the ASIS is shown in Figure 4. The top and bottom of the lap belt at the lateral location of the left and right ASIS were recorded. Points were also digitized on the belt where it crossed over the sternum and clavicle (Figure 4). In addition to the body and belt landmarks, reference points on the mockup and CRS were also recorded where applicable. The reference points allowed the body landmark data to be referenced to a seat or CRS coordinate system.

### 2.6. Posture and Belt Fit Measures

All data were expressed relative to the origin defined at the mockup seat H-point. For the highback booster conditions (B03–B06), the child data were then aligned to the ATD data obtained during ATD belt fit measurement using the booster reference points. This alignment enabled posture measures of both the child and the ATD to be computed relative to a common booster reference. For the backless booster conditions (B01–B02), the fore-aft positioning of the boosters relative to the mockup seat differed. Consequently, child and ATD posture measures were computed relative to the mockup seat H-point for these booster and mockup test conditions.

**Figure 5:**
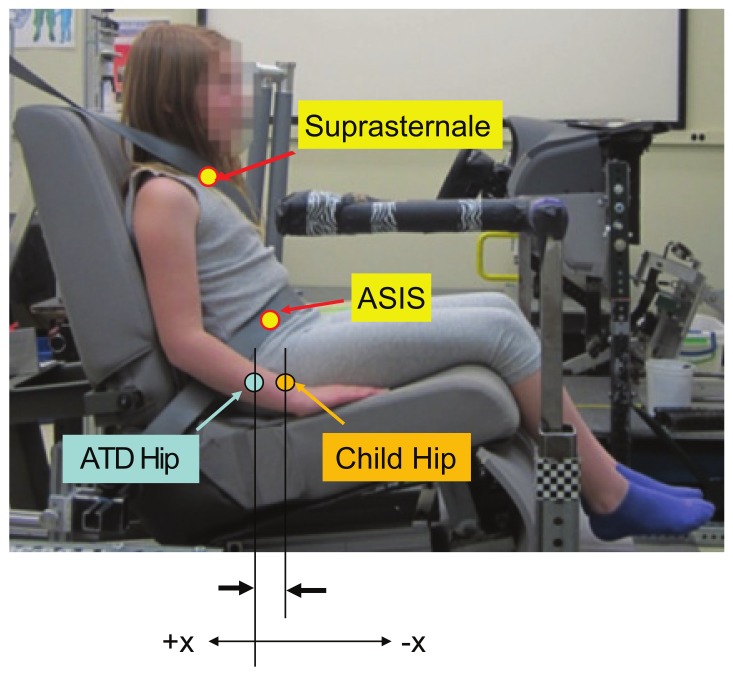
Fore-aft hip X position metric (child hip location is negative, as shown).

The posture measures were calculated to quantify differences in estimated joint-center locations between the child volunteers and the ATD. Specifically, the difference in the location of key anatomical landmarks was computed by subtracting the ATD value from the child value. The current analysis presents posture measures for the fore-aft (X) and vertical (Z) positions of the hip joint locations. Negative fore-aft X positions indicate that the child participant is forward of the ATD. Positive vertical Z positions indicate that the child is above the ATD. Figure 5 illustrates the difference between the fore-aft hip location of the child and ATD position (fore-aft hip X), for a representative child participant. Differences in head position were assessed by directly comparing the estimated location of the children’s heads’ center of gravity (headCG) with that of the 6YO ATD for each booster and mockup test condition. Head position data were expressed relative to the mockup seat H-point.

Lap and shoulder belt fit was quantified using methods developed in a previous study [24]. Belt streams were first fit with splines. The distance from the top of the lap portion of the belt to the lateral location of the anterior superior iliac spine (ASIS) landmarks on the left and right sides of the pelvis were separated into fore-aft and vertical components. Lap belt X and lap belt Z were quantified by the fore-aft and vertical locations, respectively. Right and left side measures were averaged. Lap belt X was negative if the projected belt point was forward of the ASIS. Lap belt Z was positive if the belt extended above the ASIS. The shoulder belt fit was defined as the lateral measurement between the suprasternale and the nearest point on the inner boundary of the shoulder belt at the height of the suprasternale. Positive values indicated that the belt passed to the right (buckle side) of the suprasternale landmark. This definition was chosen in part because a directly analogous measure could be obtained using the Hybrid-III 6YO ATD.

Comparisons of the mean values of posture and belt fit metrics across all booster and mockup test configurations were evaluated for statistical significance using both paired *t*-tests and the non-parametric Wilcoxon signed rank test.

## 3. Results

### 3.1. Posture Measures

**Figure 6:**
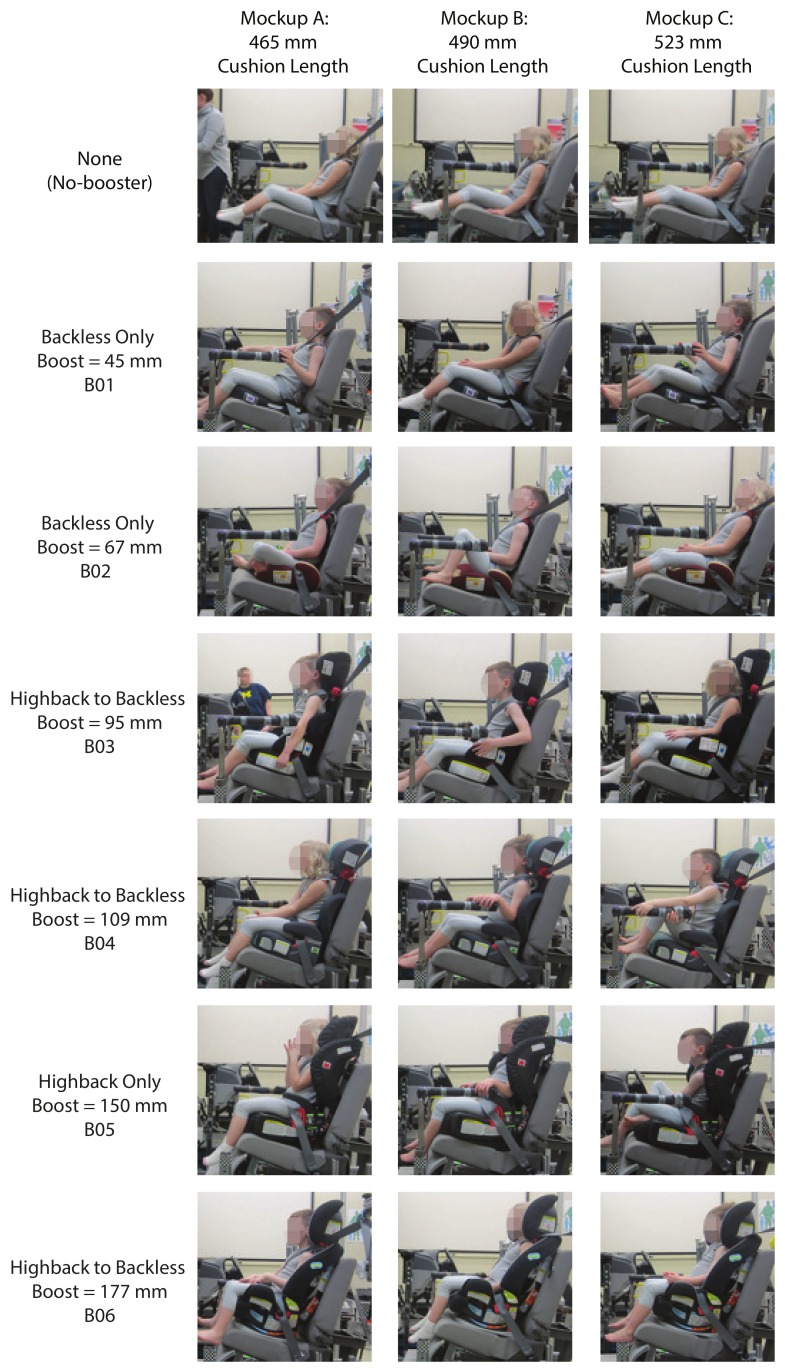
Representative child posture across the range of booster (B01–B06) configurations and mockup conditions.

Figure 6 shows the representative child participants across the range of mockup conditions tested (booster configurations and seat cushion length). Even in boosters, several volunteers chose to place their feet on the seat cushion or cross their lower extremities. Appendix A contains plots that illustrate the range of individual child postures across the booster and mockup test conditions.

#### 3.1.1. Hip Position vs. 6YO ATD

**Figure 7:**
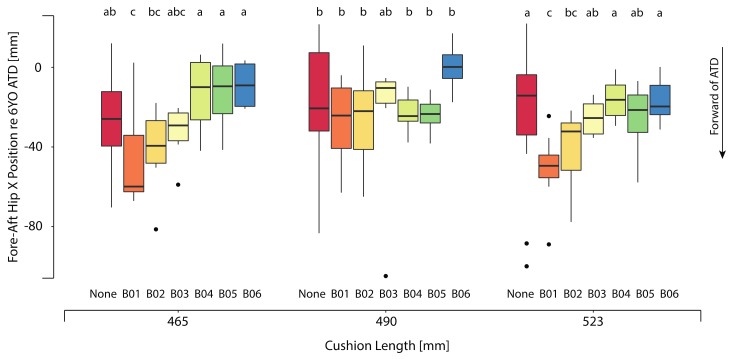
Variation of fore-aft hip X position with respect to the 6YO ATD. Negative hip X indicates that the child participant is forward of the 6YO ATD. Box plots show the median and interquartile range (25th to 75th). Whiskers extend to 1.5 * the interquartile range; outliers, defined as values exceeding 1.5 * the interquartile range, are shown as dots. Levels within a test condition that are separated by different letters are significantly different (*p* < 0.05).

Table 3 shows the mean and standard deviations of the fore-aft (hip X) and vertical (hip Z) differences between the child and ATD hip position for each booster and mockup test condition. Figure 7 shows the distribution of the calculated fore-aft hip position relative to the 6YO ATD hip X position for each booster, across the three cushion length levels. On average, the boosters that minimally boosted a child resulted in postures with the children’s hips 41 mm (B01) and 37 mm (B02) forward of the 6YO ATD. The mean fore-aft hip position of the two low-height boosters (B01 and B02) was 22 mm further forward relative to the other booster configurations. Across the cushion length conditions, children’s hip Z position was higher in comparison to the 6YO ATD by 23 mm for the no-booster condition and 11 mm for the booster with the least amount of boost (B01). For the higher boosters, the overall mean differences in the child’s vertical hip location relative to 6YO ATD were relatively small, indicating that the vertical location of the ATD hip position was representative of the child volunteers. Increasing the length of the seat cushion did not result in meaningful differences in fore-aft or vertical hip position, as shown in Table 3 and Figure 7. Figure 6 also illustrates that the children’s posture was visibly more slumped with the two lowest height boosters. The shift in posture created a gap behind the child’s pelvis.

#### 3.1.2. Head Position

**Figure 8:**
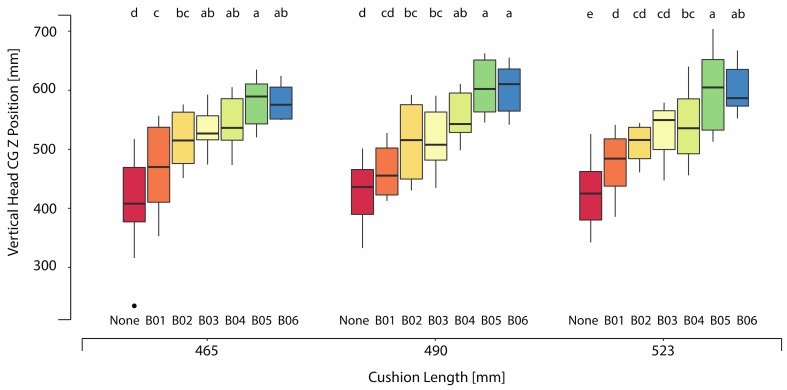
Variation of vertical headCG Z position. Positive vertical headCG Z indicates a head position higher than the ATD. Box plots show the median and interquartile range (25th to 75th). Whiskers extend to 1.5 * the interquartile range; outliers, defined as values exceeding 1.5 * interquartile range, are shown as dots. Levels within a test condition that are separated by different letters are significantly different (*p* < 0.05).

Table 4 and Figure 8 show the mean and standard deviations of the children’s fore-aft (headCG X) and vertical (headCG Z) head center of gravity location with respect to seat H-point for child participants across all booster and mockup test conditions. On average, the headCG of child participants seated in the lower height boosters (B01 and B02) were 78 mm lower and 53 mm more rearward (towards than seatback) in comparison to the higher boosters.

**Figure 9:**
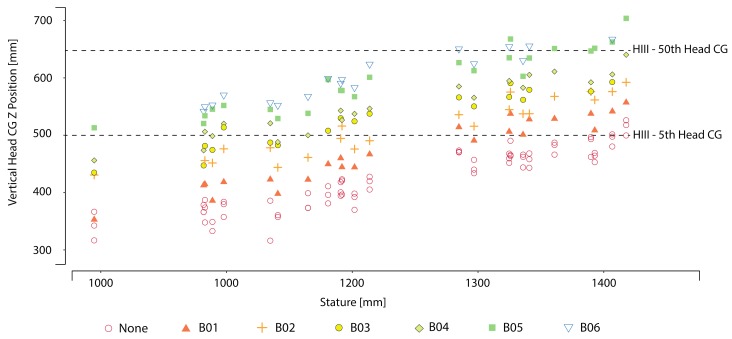
HeadCG vertical location vs. participant stature, relative to the target zone defined by the locations of small female and midsize male ATD.

Figure 9 plots the vertical head location of the child volunteers versus their stature. The dashed lines represent the range of adult ATD head vertical CG locations, defined by Hybrid-III 5th female and Hybrid-III 50th male ATD installed on the mockup seat. In the lower height boosters, only children within a stature range of 1300–1450 mm for B01 and 1250–1400 mm for B02 had their heads located within the range of adult ATD head locations.

### 3.2. Belt Fit Measures

Table 5 and Table 6 show the mean and standard deviations of the lap belt X, lap belt Z, and shoulder belt scores across the booster and mockup test conditions. The lap belt was closest to the fore-aft position of the ASIS for the no-booster and B01 booster conditions, where no booster structure was present to shift the belt relative to the pelvis. As expected, the belt extended highest above the ASIS in the no-booster condition. For most of the booster and mockup test conditions, the range of the lap belt positions was large, reflecting the variability in child belt-donning behavior. For the child participants, the shoulder belt was closest to the neck with the no-booster condition and lowest height boosters (B01), across all of the seat cushion length conditions.

## 4. Discussion

The use of belt positioning boosters reduce injury risk for child occupants compared with the use of seat belts alone. While boosters shorten the effective seat length (and thus reduce slouching), “boosting” the child relative to the vehicle interior components also achieves three additional safety benefits. First, the increase of the lap belt angle usually improves belt fit across the pelvis and reduces the risk of the occupant slipping (“submarining”) under the belt. Second, the torso belt is re-centered over the bony landmarks of the shoulder for more effective/secure restraint. Third, the child’s head is relocated in a range better protected by side airbags. Our hypothesis was that children would slouch more in lower boosters compared to taller boosters. Taller boosters effectively shorten the cushion length and enable a child’s knees to bend comfortably over the front of the booster. A low height booster may not allow the child’s knees to hang comfortably, possibly encouraging them to scoot forward to let their knees hang over the front edge of the vehicle seat. This study is the first to quantify posture and belt fit for children in booster configurations that vary widely in the amount of boost provided.

In this study, low height boosters were associated with more slouched postures, with hip positions more forward on the seat and lower head positions. Relative to the ATD, children’s hips were shifted forward more in the no-booster condition and two low height boosters vs. the taller boosters. This forward shift of the hips indicated more slumped postures. Of potential importance is the observation that posture variability among child volunteers was typically largest in the no-booster and low height booster conditions. Higher posture variability may increase the likelihood of poor restraint performance and thus greater risk of injury in the event of a crash.

Ideally, the heads of children in boosters should be positioned within the same range of adult heads. This positioning would allow children to gain the most benefit from vehicle structures and curtain airbags that are evaluated relative to adult head positions. For the current study, we considered the head locations of the small female ATD and midsized male ATD. These locations ranged from 500 to 650 mm above the H-point as possible lower and upper bounds for child head position provided by a booster. All children participating in this study fell within the allowable stature range specified by each booster, but at least one child’s head fell below the range of the adult ATD head positions in all booster conditions. Of particular interest is the fact that the low height booster left most of the children’s heads below the adult range.

Boosters improved the average lap belt fit in all mockup conditions tested. The lap belt fit scores confirmed the benefit provided by boosters, with the no-booster condition consistently having the highest lap belt placement. Additionally under this condition, the shoulder belt fell closest to the neck. Boosters B02 through B06 also shifted the belt forward compared to the no-booster and B01 condition. The low height booster (B01, which provided only 45 mm of boost), had ineffective belt guides and had belt fit measures closest to the no-booster condition. Although the low height booster (B02) only provided 75 mm of boost, it offered better lap belt fit through the design of the lap belt guides that kept the belt low and forward of the pelvis. However, the improvement in lap belt fit varied widely among all of the boosters. Both lap belt and shoulder belt fit varied across the boosters, with no observable effect of seat cushion length. Differences in lap belt measures between no-booster and booster conditions were smaller in the current study compared to those measured in past studies [10,14,15]. Since these test conditions encompassed a wider range of seat cushion lengths than in previous studies, this smaller measure may be the result of differences in parameterization of the mockup test conditions. For this study (rather than spanning the entire range of allowable angles), a range of belt angles were selected to represent the middle range seen in the vehicle fleet.

The principal limitation of this study is that all testing was conducted in a laboratory environment rather than in vehicles. Measuring child postures and belt fit in the laboratory allowed greater efficiency in testing because belt and seat geometry could be varied quickly and independently. This methodology controlled for the confounders of child occupant behavior that may be present in a naturalistic setting. Child occupant postures and behaviors have been shown to vary during everyday travel and in vehicles driven on-road [27,28,29,30]. In naturalistic driving studies (NDS) of child occupants’ head position when traveling in a CRS, Cross et al. [27] showed that children were observed to be properly positioned in their CRS for only 58% of epochs evaluated. This sampling from Cross et al. [27] was combined across forward-facing CRS and self-positioning boosters. In contrast, the results of the current study did not consider the effect on child posture from vehicle maneuvers, sleeping, or fidgeting. This study only captured “in-position” postures and belt fit and did not consider the effects of heavy clothing, which would be likely to degrade lap belt fit. Suboptimal postures and belt fit may increase the risk of injuries in the event of a crash [31]. Findings from sled tests revealed compromised safety when ATD were placed in prevalent positions observed in the NDS [31]. More specifically, suboptimal head position was associated with an increased likelihood of shoulder belt slip and greater forward head excursion.

Another limitation is that the results were based on only three seat cushion lengths obtained with a single vehicle seat mockup. Different vehicle seat and belt designs could change the relative performance of the boosters. Due to the relatively small sample of volunteers, no statistically significant differences in belt fit were observed between booster configurations. However, based on previous studies, these differences would likely be small in absolute terms.

## 5. Conclusions

This study is the first to quantify posture and belt fit for children in booster configurations that vary widely in the amount of boost provided. Low height boosters produced postures that were more slouched (hips further forward) than in other typical boosters. Belt fit in the low height boosters was not meaningfully different from the other boosters. Dynamic performance is what ultimately matters, but good static posture and belt fit are crucial pre-conditions for robust crash protection.

## Figures and Tables

**Table 1:** Mockup belt anchorage conditions compared to fleet measurements [22].

D-Ring Location Levels	*X*	*Y*	*Z*	Side-View (*XZ*) Angle (deg)	Front-View (*YZ*) Angle (deg) *
Fleet −1 STD	309	210	510	26	20
Fleet +1 STD	513	298	652	44	28
Mockup A	410	200	540	37	20
Mockup B	350	270	610	30	24
Mockup C	474	244	588	39	23
**Lower Anchorage Location Levels**	***X***	***Y***	***Z***	**Side-View (*XZ*) Angle (deg) †**	**Front-View (*YZ*) Angle (deg)**
Fleet −1 STD	54	214	−83	40	18
Fleet +1 STD	164	320	−207	66	38
Mockup A	130	250	−160	51	37
Mockup B	60	220	−200	73	42
Mockup C	155	225	−137	41	31

* D-ring *XZ* and *XY* Angle with respect to vertical. † Lap belt angle with respect to horizontal.

**Table 2:** Participant characteristics stratified by participant group.

	Group 1			Group 2			Group 3		
	(4 Boys, 5 Girls)			(4 Boys, 4 Girls)			(5 Boys, 3 Girls)		
Measurement	Mean	Min	Max	Mean	Min	Max	Mean	Min	Max
Age (year)	7	4	9	7	4	10	7	4	11
Stature (mm)	1241	993	1416	1222	1080	1339	1251	1087	1405
Weight (kg)	26.9	15.5	53.9	25.0	19.5	35.2	27.5	17.4	41.7
Erect Sitting Height (mm)	666	567	765	651	594	693	664	592	726
Body Mass Index (kg/m^2^)	17.1	14.3	30.7	16.6	14.2	19.8	17.2	14.5	21.2

**Table 3:** Mean (SD) of the fore-aft (hip X) and vertical (hip Z) hip position, expressed with respect to the 6YO ATD. Negative hip X indicates that the child participant is forward of the 6YO ATD. Positive hip Z indicates that the child participants’ hip position is above the 6YO ATD. Pairwise comparison (PW) of the levels within a test condition that are separated by different letters are significantly different (*p* < 0.05).

Booster	Fore-Aft Hip X Position						Vertical Hip Z Position					
	re 6YO ATD (Hip X)						re 6YO ATD (Hip Z)					
	Mockup A		Mockup B		Mockup C		Mockup A		Mockup B		Mockup C	
	Mean (SD)	PW	Mean (SD)	PW	Mean (SD)	PW	Mean (SD)	PW	Mean (SD)	PW	Mean (SD)	PW
**None**	−27 (24)	ab	−18 (29)	b	−20 (29)	a	23 (18)	a	19 (14)	a	26 (21)	a
**B01**	−46 (26)	c	−27 (22)	b	−51 (19)	c	12 (15)	ab	8 (8)	bc	14 (11)	ab
**B02**	−41 (20)	bc	−26 (26)	b	−42 (21)	bc	4 (11)	b	1 (17)	bc	9 (10)	bc
**B03**	−33 (14)	abc	−11 (6)	ab	−25 (9)	ab	−0 (20)	b	−5 (10)	c	6 (9)	bc
**B04**	−13 (19)	a	−23 (10)	b	−16 (11)	a	−2 (4)	b	2 (17)	bc	−9 (15)	bc
**B05**	−12 (20)	a	−24 (10)	b	−25 (17)	ab	8 (9)	b	11 (7)	ab	7 (16)	c
**B06**	−9 (13)	a	0 (11)	a	−17 (12)	a	0 (9)	b	0 (7)	bc	0 (10)	bc

**Table 4:** Mean (SD) of the fore-aft (headCG X) and vertical (headCG Z) head center of gravity position, expressed with respect to the 6YO ATD (mm). Positive fore-aft headCG indicates a child head location rearward closer to the seatback than the ATD head. Positive vertical headCG indicates a head position higher than the ATD. Pairwise comparison (PW) of levels within a test condition that are separated by different letters are significantly different (*p* < 0.05).

Booster	Fore-Aft HeadCG X Position						Vertical HeadCG Z Position					
	Mockup A		Mockup B		Mockup C		Mockup A		Mockup B		Mockup C	
	Mean (SD)	PW	Mean (SD)	PW	Mean (SD)	PW	Mean(SD)	PW	Mean (SD)	PW	Mean (SD)	PW
**None**	145 (25)	a	144 (35)	bc	150 (37)	a	415 (69)	d	429 (51)	d	420 (51)	e
**B01**	156 (28)	a	187 (38)	a	159 (33)	a	467 (75)	c	462 (44)	cd	475 (57)	d
**B02**	160 (21)	a	159 (25)	ab	155 (44)	a	517 (49)	bc	513 (21)	bc	509 (33)	cd
**B03**	108 (55)	b	133 (25)	bcd	118 (58)	abc	533 (41)	ab	505 (51)	bc	530 (52)	cd
**B04**	147 (26)	a	119 (50)	cde	145 (54)	ab	545 (47)	ab	557 (44)	ab	541 (70)	bc
**B05**	97 (34)	b	90 (26)	e	96 (51)	c	582 (44)	a	604 (49)	a	600 (70)	a
**B06**	126 (15)	ab	113 (16)	de	105 (20)	bc	581 (37)	ab	603 (44)	a	602 (46)	ab

**Table 5:** Mean (SD) lap belt X and Z. Negative lap belt X indicates that the belt is forward of the projected ASIS location. Positive lap belt Z indicates that the upper edge of the belt lies above the ASIS. Pairwise comparison (PW) of levels within a test condition that are separated by different letters are significantly different (*p* < 0.05).

Booster	Lap Belt X						Lap Belt Z					
	Mockup A		Mockup B		Mockup C		Mockup A		Mockup B		Mockup C	
	Mean (SD)	PW	Mean (SD)	PW	Mean (SD)	PW	Mean (SD)	PW	Mean (SD)	PW	Mean (SD)	PW
**None**	−15 (11)	a	−5 (14)	a	−2 (15)	a	19 (10)	a	24 (9)	a	27 (12)	a
**B01**	−10 (14)	ab	−12 (12)	ab	−11 (13)	ab	13 (9)	ab	12 (11)	b	13 (8)	bc
**B02**	−26 (19)	c	−28 (5)	d	−28 (12)	c	8 (9)	b	9 (10)	b	7 (8)	c
**B03**	−29 (13)	c	−26 (10)	cd	−25 (14)	c	14 (10)	ab	9 (12)	b	19 (9)	ab
**B04**	−26 (18)	bc	−33 (7)	d	−25 (9)	c	12 (11)	ab	13 (8)	b	11 (5)	bc
**B05**	−26 (10)	bc	−27 (10)	cd	−28 (7)	c	10 (7)	b	14 (9)	b	16 (12)	bc
**B06**	−23 (13)	abc	−16 (11)	bc	−20 (9)	bc	9 (7)	b	11 (8)	b	17 (11)	bc

**Table 6:** Mean (SD) shoulder belt fit. Positive shoulder belt fit values indicate that the belt passes to the right (buckle side) of the suprasternale landmark. Pairwise comparison (PW) of levels within a test condition that are separated by different letters are significantly different (*p* < 0.05).

Booster	Shoulder Belt Fit					
	Mockup A		Mockup B		Mockup C	
	Mean (SD)	PW	Mean (SD)	PW	Mean (SD)	PW
**None**	−9 (15)	e	−7 (16)	e	−13 (15)	b
**B01**	−3 (18)	e	6 (13)	de	−3 (16)	b
**B02**	67 (18)	a	31 (15)	abc	36 (11)	a
**B03**	64 (30)	ab	51 (49)	a	35 (16)	a
**B04**	20 (10)	d	24 (12)	bcd	22 (27)	a
**B05**	47 (17)	bc	43 (24)	ab	34 (21)	a
**B06**	30 (31)	cd	18 (8)	cd	34 (26)	a

**Figure A1:**
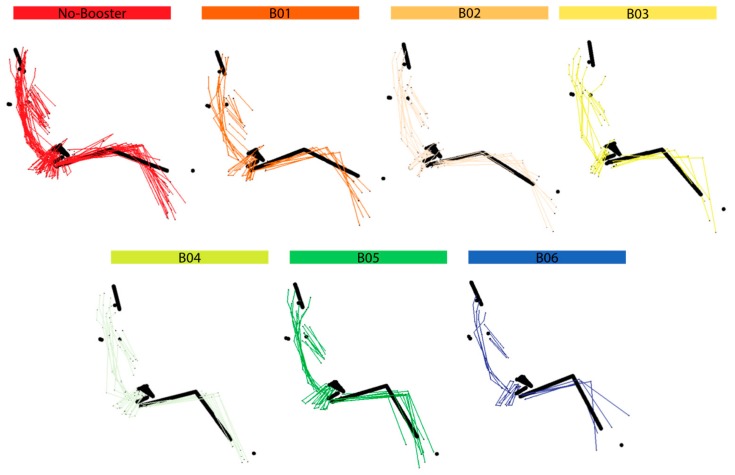
Comparison of postures of child volunteers overlaid and expressed with respect to 6YO ATD across all booster conditions in Mockup A. Cushion length = 465 mm; seat back angle = 23 deg; cushion angle = 14.5 deg.

**Figure A2:**
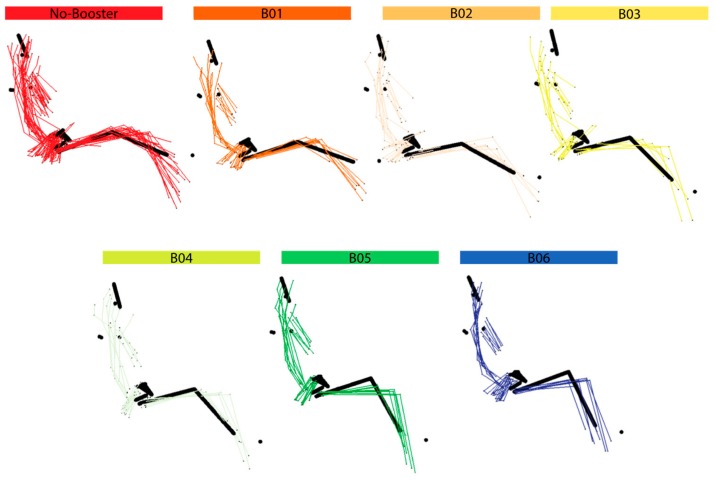
Comparison of postures of child volunteers overlaid and expressed with respect to 6YO ATD across all booster conditions in Mockup A. Cushion length = 490 mm; seat back angle = 23 deg; cushion angle = 14.5 deg.

**Figure A3:**
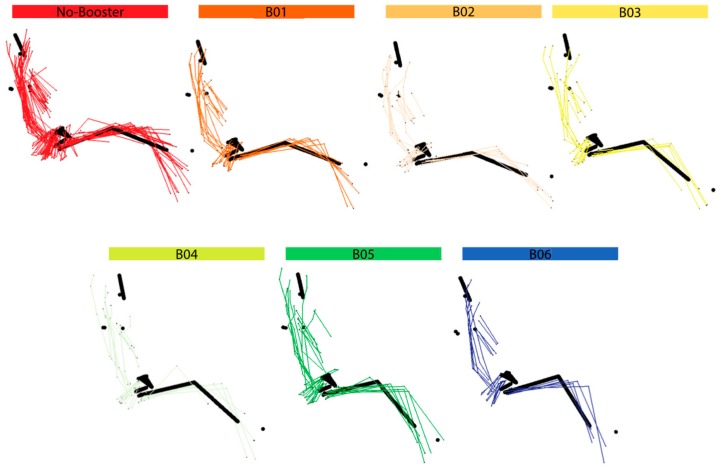
Comparison of postures of child volunteers overlaid and expressed with respect to 6YO ATD across all booster conditions in Mockup A. Cushion length = 523 mm; seat back angle = 23 deg; cushion angle = 14.5 deg.
